# Local and Systemic Oxidative Stress in Balkan Endemic Nephropathy Is Not Associated with Xanthine Oxidase Activity

**DOI:** 10.1155/2020/8209727

**Published:** 2020-08-18

**Authors:** Andrej Veljković, Jovan Hadži- Đokić, Dušan Sokolović, Rade Čukuranović, Jovana Čukuranović-Kokoris, Dragoslav Bašić, Branka Đorđević, Marko Stojanović, Andrija Šmelcerović, Gordana Kocić

**Affiliations:** ^1^Faculty of Medicine, University of Nis, 18000 Nis, Serbia; ^2^Serbian Academy of Sciences and Arts, 11000 Belgrade, Serbia; ^3^Clinical Center in Nis, 18000 Nis, Serbia

## Abstract

Balkan endemic nephropathy (BEN) represents a chronic tubulointerstitial nephropathy which is followed by the progression of kidney fibrosis to end-stage kidney failure. The critical involvement of poisons in food (aristolochic acid (AA), ochratoxin, and heavy metals) and selenium deficiency are among nutritive factors which contribute to the pathogenesis of BEN, due to reactive oxygen species (ROS) liberation and/or decreased antioxidative defence system. The aim of the study is to distinguish a possible systemic and local origin of ROS through the measurement of xanthine oxidase (XO) activity in urine and plasma, along with the determination of the oxidative changes in lipids and proteins. The study included 50 patients with BEN and 38 control healthy subjects. We noted increased levels of both thiobarbituric acid-reactive substances (TBARS) and advanced oxidation protein products (AOPPs) in the plasma of patients with BEN, compared to the control group (*p* < 0.001). The urinary levels of AOPPs were higher in patients with BEN in comparison to the control (*p* < 0.001). The specific activity of XO was significantly lower in plasma and urine in BEN samples, compared to controls (*p* < 0.005). Based on these results, we hypothesize that XO might not be considered a direct systemic or local contributor to ROS production in BEN, most probably because of the diminished kidney functional tissue mass and/or AA-induced changes in purine nucleotide conformation. The increased AOPP and TBARS level in both plasma and urine in BEN may predict ROS systemic liberation with toxic local effects.

## 1. Introduction

Balkan endemic nephropathy (BEN) is a chronic tubulointerstitial nephropathy characterized by an unpredictable onset and a gradual progression to end-stage renal disease. Upper urothelial cancer (UUC) of the pelvis and ureter has increased prevalence in BEN patients [1–3] and predisposes to increased mortality risk [4]. Moreover, it has been reported that kidney transplantation in BEN patients, although partially restores renal function, does not reduce the risk of developing UUC [5]. This disease was first discussed over sixty years ago [6, 7]. Typical of the disease is that it affects residents of villages of certain areas of the Danube tributaries within the region of the Balkans [8, 9].

The pathophysiology of BEN has been elaborated in a number of reports [9–11]. It is similar to all tubulointerstitial nephritic diseases and is characterized by progressive kidney atrophy and sclerosis [9]. Histopathological findings indicate a hypocellular interstitial fibrosis accompanied with tubular atrophy. Glomerular and vascular lesions are associated with periglomerular fibrosis, ischaemic, microcystic glomeruli, thrombotic microangiopathy-like lesions, and focal segmental sclerosis-like lesions [9]. To date, the aetiology of BEN is not yet fully elucidated. Some suggest that environmental factors might play a pivotal role in its pathophysiology, while others support that confounding factors might also be involved. Among these, the chronic food poisoning by aristolochic acid (AA), a toxin present in plants of the genus Aristolochia, seems to be the most acceptable theory. AA promotes kidney damage in BEN, associated with the hepatic enzymes metabolizing AA, which may have high cancerogenic potential [12–14]. A prolonged exposure to AA-contaminated food grown in polluted soil could be one of the main aetiological mechanisms of BEN observed in the Balkan area. Since AAs are resistant to degradation, it is possible that they could be absorbed by the root and transported to the edible part of food crops [15]. Metabolic activation of AAs leads to a reduction of the nitro group to produce N-hydroxylaristolactams (N-hydroxyl-ALs) [16–20]. Hydrolysed lactams create reactive nitrenium ions which yield large AL-purine deoxyribonucleic acid adducts (addition products) at the exocyclic amino groups of the purines. The foremost overabundant deoxyribonucleic acid adduct, 7-(deoxyadenosine-N6-yl) aristo-lactam I, causes characteristic AT→TA transverse found in the gene responsible for tumour-suppressive protein p53 transcription [19, 20]. Furthermore, mycotoxins such as ochratoxin (OTA) and citrinin, which are mainly produced by some species of Penicillium, Aspergillus, and Monascus genera, are implicated to be a potential cause of BEN [21]. The concentrations and the extent of selenium deficiency are documented in rock, soil, water, foodstuff, and blood samples collected from endemic and nonendemic regions of BEN in the geographical area of Serbia [22–24]. Some findings suggest that heavy metals and metalloids may also contribute to BEN or similar diseases in the same geographic area [25].

Increased oxidative stress (OS) caused by overproduction of reactive oxygen species (ROS) and deficiency of antioxidative defence systems may be possible common mechanisms for all of the factors mentioned above, including the progressive renal injury of the disease [26]. Moreover, OS might also contribute to the increased cardiovascular burden seen in CKD patients [27]. Accumulation of ROS starts at early CKD stages and is gradually elevated, parallel to progression to end-stage renal disease [28]. Also, all tubulointerstitial injuries are characterized by the generation of free oxygen radicals, highly nephrotoxic and very reactive metabolites, released throughout the oxidative burst [29]. ROS may indirectly cause the formation of DNA adducts by initiating autocatalytic lipid peroxidation, which generates a large variety of potential genotoxic breakdown products, including alkoxy radicals, peroxyl radicals, and aldehydes, such as thiobarbituric acid-reactive substances (TBARS) [30]. Lipid peroxidation is one of the most significant implications of ROS generation, which causes irreversible damage to the function and structure of cell membranes. TBARS, the final products of OS, might also serve as indicators of lipid peroxidation status [31]. Moreover, ROS can also cause irreversible damage in the morphology and function of proteins, carbohydrates, and DNA. Earlier studies have found that plasma concentration of advanced oxidation protein products (AOPPs) significantly increased with the progression of nephritic pathology in CKD patients [32] and in patients with diabetes [33]. To date, the exact pathophysiologic mechanism underlying the overproduction of ROS has not yet been elucidated. However, accelerated inflammation and impaired antioxidative defence mechanisms have been in the centre of scientific attention. OS might also mediate urinary tract injury through direct cytotoxic effects on proximal tubular cells and altered renovascular responses or even as a configuration of many factors.

One of the hypothetical mechanisms for ROS production in BEN may be the xanthine oxidase (XO) reaction. XO is the oxidative radical-forming isoform of xanthine oxidoreductase. XO is the main enzyme involved in uric acid production, acting as the final metabolite of the adenine nucleotides. Simultaneously with the production of uric acid, XO activity liberates hydrogen peroxide and superoxide anion, well-established prooxidant molecules [34]. Increased plasma XO activity has been reported in several disease states, such as cholecystitis, shock, ischaemia-reperfusion injury, acute virus infection, adult respiratory distress syndrome, and carcinogenesis [28]. However, it remains debatable whether XO activity is involved in the pathogenesis of BEN-associated kidney injury and cancerogenesis of the upper urothelial tract.

The aim of the present study is to determine whether OS is involved in the pathogenesis of BEN, observed via oxidative changes in lipids and proteins in plasma and urine.

## 2. Materials and Methods

All the reagents were purchased from Sigma (St. Louis, MO, USA). All chemicals used were of analytical grade.

### 2.1. Patients

We recruited patients with BEN from the Institute of Nephrology, Clinical Centre of Nis, Serbia, in accordance with a standard diagnostic protocol [35]. All subjects gave their informed consent before they participated in the study. The study was conducted in accordance with the Declaration of Helsinki, and the protocol was approved by the Ethics Committee of Clinical Centre in Niš (Decision No. 17776/8). Regarding the tubulointerstitial character of the disease, the clinical-biochemical diagnostic set used the following: (1) a history of the birth place and their place of residence; (2) laboratory urine analysis: glomerular filtration rate (GFR) reduction, which was less than 60 ml/min/1.73 m^2^ in these patients; microalbuminuria (proteinuria < 1 g/24 h) associated with urinary marker tubular lesions (*β*2-microglobulin in urine), laboratory blood test: urea and creatinine; (3) radiological findings; and (4) kidney ultrasound. The estimates included in the study [36] were selected from the endemic areas around South Morava. Patients with BEN who had other chronic diseases were excluded from the study. The control group [37] involved the corresponding non-BEN rural areas. The control patients did not have any acute or chronic illness or high blood pressure, since hypertension is not the characteristic of BEN. The respondents were matched for gender and age. The demographic characteristics of BEN patients and the control group are shown in Table 1. Blood and urine of BEN patients and the control groups were collected, centrifuged to separate plasma at 3000 rpm, and stored at -20°C prior to the analysis.

### 2.2. Methods

We analysed plasma and urine for biochemical parameters on an A24 automatic analyser for in vitro diagnostics (Biosystems SA).

#### 2.2.1. Lipid Peroxidation Products

Lipid peroxidation in urine and plasma in terms of TBARS formation was determined using a slightly modified method of Nabavi et al. [38]. We added TCA and TBA into the serum. Subsequently, it was incubated at 100°C for one hour. After cooling, the samples were centrifuged to remove the precipitate, the supernatant is separated, and the quantity of TBA-reactive lipid peroxidation products was measured at 532 nm against a blank which contained all the reagents except the serum. The concentration of TBARS is expressed in *μ*mol/l.

#### 2.2.2. AOPP Concentration

The concentration of AOPPs in plasma and urine was determined by spectrophotometric technique according to the method of Witko-Sarsat et al. [32]. We diluted 200 microliters of plasma in 1 : 5 in PBS or chloramine-T standard solutions, which were placed in wells of a 96-well microtiter plate, to which 20 *μ*l of acetic acid was added. Ten microliters of 1.16 M potassium iodide was then added, followed by 20 *μ*l of acetic acid. The absorbance of the mixture was immediately read at 340 nm in a microplate reader against a blank containing all reagents only. The AOPP concentration is expressed in *μ*mol/l chloramine-T.

#### 2.2.3. XO Activity

The specific activity of XO was evaluated in plasma, spectrophotometrically, according to the liberation of uric acid by using xanthine as substrate, in the absence of NADH in cases in which only molecular oxygen was the acceptor of electrons. Uric acid was stoichiometrically formed from xanthine, and it was measured at 293 nm. XO activity was expressed in IU/l [37].

### 2.3. Statistical Analysis

Quantitative variables were presented as arithmetic means with standard deviation (mean ± SD). Differences between the group means were analysed using one-way analysis of variance. *p* value < 0.05 was accepted as statistically significant. We performed the statistical analysis with Statistical Package for the Social Sciences for Windows (SPSS, version 11.0, Chicago, Illinois, USA).

## 3. Results

**Figure 1:**
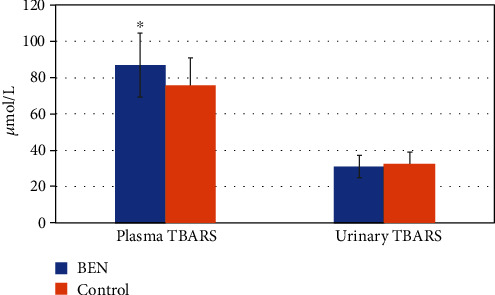
TBARS values in patients with BEN and controls. Data are mean ± SD values. ∗*p* < 0.05 versus control.

**Figure 2:**
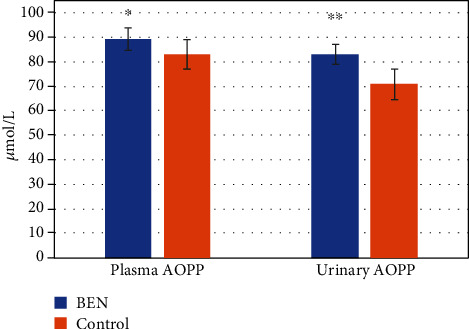
AOPP values in BEN patients and controls. Data are mean ± SD values. ∗*p* < 0.05 versus control; ∗∗*p* < 0.001 versus control.

**Figure 3:**
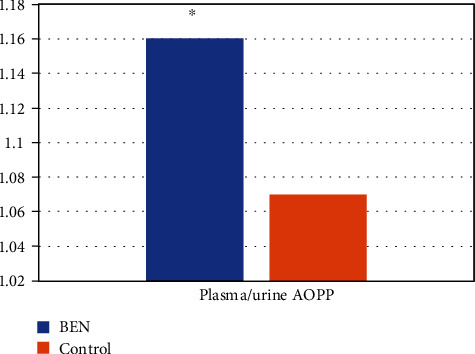
AOPP values in patients with BEN and controls: plasma/urine ratio. ∗*p* < 0.001 versus control.

**Figure 4:**
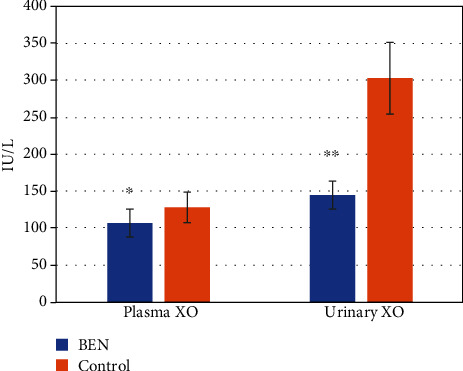
Xanthine oxidase activity in patients with BEN and controls. Data are mean ± SD values. ∗*p* < 0.05 versus control; ∗∗*p* < 0.001 versus control.

**Figure 5:**
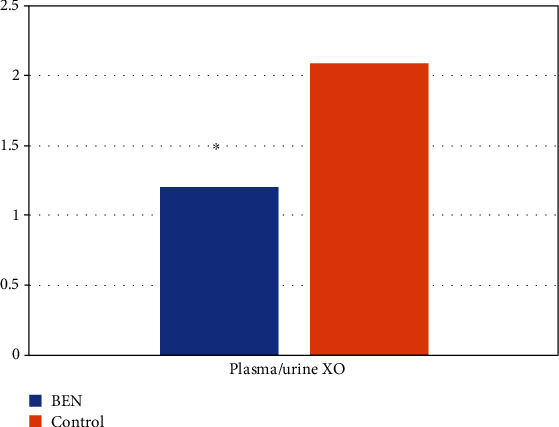
XO values in patients with BEN and controls: plasma/urine. ∗*p* < 0.001 versus control.

The concentration of TBARS in plasma and urine is shown on Figure 1. Compared to controls, BEN patients exhibited significantly increased plasma TBARS levels (*p* < 0.001). However, urine TBARS levels were not significantly different among groups. Therefore, compared to healthy controls, BEN patients present an increased systemic lipid peroxidation status. However, the local, urine lipid oxidation status did not differ among groups, presumably due to the low lipid content in the urine. The level of AOPP in plasma and urine is shown in Figure 2, whereas Figure 3 shows the difference in plasma/urine AOPP ratio among groups. Compared to those in controls, plasma and urine levels of AOPPs and plasma/urine AOPP ratio were significantly increased in BEN patients (*p* < 0.05, *p* < 0.001, and *p* < 0.001, respectively). Therefore, the damaging effect of OS on proteins is significantly pronounced in BEN patients both systemically (in plasma) and locally (in urine), compared to healthy individuals. The activity of XO in plasma and urine is shown in Figure 4. The decrease in plasma XO activity of BEN patients was statistically significant when compared to that of controls (*p* < 0.05). We can assume that there is a systemic lower activity of XO. The urinary level of XO activity was significantly lower in patients with BEN when compared to the control group (*p* < 0.001), which suggests a local low XO activity. Moreover, we found statistically significant lower plasma/urine XO ratio in BEN patients when compared to controls (*p* < 0.001) (Figure 5). We hypothesize that there is a low systemic level of XO activity in patients with BEN.

## 4. Discussion

BEN affects a large number of inhabitants of endemic areas. In the last decades, epidemiologic findings have revealed that BEN is an environmentally induced disease. Aristolochia, ochratoxin, selenium deficiency, and heavy metals are among the most important factors contributing to the onset and development of this disorder. However, the influence of these factors in association with chronic tubulointerstitial nephropathy and end-stage renal disease remains undetermined.

The association of food containing AA with BEN and UUC was initially documented in 1969 by Ivić [39]. Also, the inhabitants in regions characterized by BEN are exposed to relatively high concentrations of OTA [40].

Heavy metals are also implicated in the pathophysiology of BEN. Among them, increased levels of silica, lead, uranium, copper, cobalt, zinc, manganese, arsenic, titanium, barium, aluminium, chromium, strontium, cadmium, bismuth, molybdenum, nickel, tungsten, and antimony in water and soil have been documented in areas with increased incidence and prevalence of BEN [13]. It is well known that metal ions can produce free radicals via Fenton reaction. We documented the influence of a heavy metal intake and ROS development in our previous study [41]. The concentrations and the extent of selenium deficiency (a well-known antioxidant) have also been documented in rock, soil, water, foodstuff, and blood serum samples collected from endemic and nonendemic regions of BEN in the geographic area [22–24].

The deoxyribonucleic acid DNA-AA adducts induced by AA produce a particular molecular signature in the kidneys with BEN nephropathy. However, the pathophysiological mechanism whereby AA results in renal injury is still unclear. Declèves et al. [42] showed that nitric oxide (NO) plays an important role in mediating AA-induced kidney injury, leading to enhanced OS and tubular cell programmed cell death. AA-induced OS could mediate a peritubular capillary loss and additional vascular transformation, the vital pathophysiological processes within the development of chronic nephropathy from acute kidney injury in different injury models.

Among mycotoxins, OTA was documented to be an OS inducer [40]. Taking into consideration all the information, the EU Food Safety Authority (EFSA) scientific panel on contaminants within the food chain concluded that there was no proof for the existence of specific OTA-DNA adducts and that the genotoxic effects of OTA were presumably the result of OS injury [43].

The kidneys are extremely vulnerable to ROS damage, because of long-chain-polyunsaturated fatty acids present in cell membranes and higher local concentration of excretory toxic products. The results of our study demonstrated a higher plasma level of TBARS in patients with BEN compared to controls. One of the first events in oxidative cellular injury is the oxidization of membrane lipids. Lipid hydroperoxides are nonradical intermediates derived from unsaturated fatty acids, phospholipids, glycolipids, cholesterol esters, and cholesterol itself. Their formation occurs in an enzymatic or nonenzymatic reaction involving ROS. Measuring a breakdown product like TBARS, a nephrotoxic molecule which is a biological marker of OS, is the most frequently performed method for determination of lipid peroxidation [44]. In our study, the increased level of lipid peroxidation was statistically significant in plasma, but not in urine, which may imply that systemic OS may arise due to kidney dysfunction and accumulation of different uremic toxins. A recently published article by Kim et al. [45] has shown that the administration of melatonin as a well-known antioxidant interfered with AA-induced renal dysfunction, suppressed AA-induced OS, as evidenced by the downregulation of 4-hydroxynonenal and reduced level of malondialdehyde and a modulated expression of prooxidant and antioxidant enzymes.

Circulating AOPPs serve as a marker of protein oxidation status. Our study showed that AOPPs were significantly elevated both in the plasma and urine of patients with BEN. Li et al. [46] reported that in the remnant kidney model, a higher AOPP level resulted in accelerated progression of renal injury, as proved by a marked increase of tubular fibrosis and glomerulosclerosis, additionally higher level of albuminuria, and deteriorated renal dysfunction. Moreover, experimental AOPP administration raised the urinary protein excretion in sham-operated rats thus confirming their direct toxic effect [46]. Our results also suggest that AOPPs are involved in the process of renal recast, because of the subsequent fibrogenic process in the kidney. Furthermore, the increase in the plasma/urine ratio in BEN patients might indicate their systemic origin as well. Li et al. [46] reported that chronic administration of AOPPs during this remnant kidney model raised the renal levels of AOPPs considerably, followed by magnified levels of TBARS and a reduced Glutathione Peroxidase (GSHPx) activity. Moreover, exogenous AOPP administration evoked an imbalance of oxidation-reduction reaction in rats with intact kidney and normal renal function, suggesting that a more severe OS could not *per se* promote the progression of kidney dysfunction. In sum, these results suggest that AOPPs could be one of the inducers of OS in BEN. A close relationship between AOPP and TBARS levels or GSHPx activity and the in vitro studies demonstrating a respiratory burst of human neutrophils which were exposed to AOPPs give additional proof to support the notions [47]. Since chronic AOPP administration raised the expression of transforming growth factor beta 1 (TGF-*β*1), a well-documented fibrogenic growth factor, there was a direct connection between the AOPP and the pathogenesis of nephritic inflammation and fibrosis, which is one of the key mechanisms of kidney deterioration in patients with BEN [48].

ROS can directly damage the deoxyribonucleic acid of proximal tubular cells and accelerate programmed cell death. To support this hypothesis, it was reported in a previous study that AA depleted the antioxidant glutathione in human renal tubular cells (HK-2) leading to tubular cell death [36]. Tubular cell death may be related to the OS-induced vascular effects of AA. Intrarenal OS exacerbates smooth muscle cell proliferation of the afferent arterioles and promotes renin-angiotensin system activation. It was documented that ischaemic injury as a part of AA-induced acute kidney injury is related to reduced NO levels; such OS-induced vascular changes may also lead to tubular cell death. AA can directly reduce the concentration of NO and can additionally cause the peritubular capillary loss and vascular transformation [49]. Another important question is which is the most probable source of ROS overproduction. AA may be a direct inducer of OS, but that is certainly not the main cause of it. In the literature, there is extensive evidence regarding the role of enhanced ROS within the kidney and protective role of antioxidants and ROS scavengers in ischaemia-reperfusion injury [49–52]. Indeed, fibrotic kidney is under ischaemia. However, this mechanism occurs only in the last stages of the disease.

Another potential source of ROS could be uremic toxins in patients with CKD. A higher level of those toxins promotes systemic inflammation via priming polymorphonuclear leukocytes and stimulating CD-8^+^ cells [53]. Increased OS occurs even in early stages of the disease, progresses with deterioration of renal function, and is further aggravated by hemodialysis (HD), due to the bioincompatibility of HD systems [54, 55]. In general, two major parts of a HD system will contribute to OS: the dialyzer membrane and trace endotoxins within the dialysate [56]. However, it is an unlikely explanation in this disease because not all patients with BEN were uremic.

Our hypothesis was that the source of free radicals could be XO activity, since the liver, gut, and kidney represent XO-rich organs. XO-induced ROS production is known to be elevated in septic development by significant liver and kidney injuries, and XO inhibitors are one of the protectors from kidney injuries [57]. Additionally, the long-term treatment with XO inhibitors achieved renoprotective advantages in individuals with a nonadvanced chronic renal disorder [58]. A large body of clinical proof has cited uric acid as a possible therapeutic target for slowing down CKD progression [59]. Increased serum levels of uric acid have been associated with the onset and development of chronic kidney disease (CKD), cardiovascular disease, and mortality, through several molecular pathogenetic mechanisms, such as inflammation and OS [60]. Gouty patients and even people with symptomless hyperuricemia have a sustained risk of developing renal damage; equally, in patients with obvious CKD, steady elevated uric acid levels might contribute to the deterioration of renal function [61]. The deterioration of renal function which leads to end-stage disease such as BEN might be the result of coexisting conditions such as vascular calcification, obesity, and hypertension and not only elevated serum uric acid [62]. Since ROS are closely related to kidney pathology [63], it has been reported that XOR redox, i.e., the quantitative relation of XO to total XOR (XO and XDH), changes the oxidative state and is in correlation with kidney pathology [34]. We find XOR not only as a xanthine dehydrogenase (XDH) type but also as an XO form, which uses an oxygen molecule as an electron acceptor and generates ROS as superoxide anion radicals and hydrogen peroxide [34]. We have already proven that XO activity is one of the possible reasons for ROS liberation in experimental kidney damage [41]. It is also demonstrated that XO is responsible for ischaemia injury and fibrosis [64].

All findings mentioned above led to the expectancy of a higher activity of XO in BEN patients since there were many causes which would raise its activity, but this was not the case. Our results showed a lower XO activity in patients with BEN when compared to healthy control subjects. Plasma/urine ratio showed even more significant differences, which may point to a systemic effect of the enzyme. There are only a few patients with BEN who suffered from gout. One of the possible reasons would be the lack of actual substrates for XO, like urine nucleotides. Lower kidney mass, accompanied with diminished functional tissue, may be responsible for a lower substrate level. Furthermore, studies showed the importance of AA in the formation of DNA-AA adducts [16]. The resulting adenine (AL-N6-dA) and guanine (ALN2-dg) adducts misincorporate adenine throughout replication [20, 21]. Specifically, the intrinsic twisted conformation of ALII-N2-dG induces destabilizing distortions to deoxyribonucleic acid at the lesion site, reduces van der Waals (stacking) interactions with the neighbouring base pairs, and enhances the helical dynamics at the damaged site [65]. The abovementioned changes affect the purine nucleotide structure in patients with BEN, probably making them conformationally not recognizable for XO, because AA binds covalently with the exocyclic amino group of purine nucleotides.

## 5. Conclusions

The level of lipid peroxides and AOPPs is significantly increased in BEN patients compared to controls. We hypothesize that both local and systemic overproduction of ROS might play a pivotal role in the pathogenesis of BEN which leads to end-stage renal disease and even cancerogenesis. Our results may for the first time demonstrate that XO would not be considered a direct systemic or local contributor to ROS production in BEN, most probably because of the diminished kidney functional tissue mass and AA-induced changes in purine nucleotide conformation. A low XO activity might also prevent gout in patients with BEN.

## Figures and Tables

**Table 1:** Baseline characteristics of BEN study cases and control group.

Characteristics	BEN	Controls	*p* value
Men	30 (60%)	22 (57.5%)	0.866
Women	20 (40%)	16 (42.5%)	
Age (years)	72 (52.6-86.7)	73 (65.05-83.95)	0.377
SCr (mol/l)	120.2 (70.22-606.4)	80.2 (68.63-125.15)	*0.001*
CCr (ml/min)	35.61 (7.93-87.41)	65.9 (23.09-108.7)	*0.001*
UCr (mmol/l)	7.02 (1.77-22.75)	10.23 (5.2-23.53)	*0.020*
UPCr (mg/mmol)	20.61 (5.43-423.47)	10.42 (5.22-24.24)	*0.001*
UACr (mg/mmol)	1.22 (0.18-60.72)	0.87 (0.2-12.07)	*0.043*
Hgb (g/l)	13.1 (8.7-131.6)	122 (12.71-152.9)	*0.001*
Glucose (mmol/l)	4.66 (3.8-6.4)	5.3 (4.31-6.89)	*0.022*
*β*2-Microglobulin (g/l)	94.81 (5.85-4754)	—	—
U protein (mg/l)	215 (36.5-1510.5)	107 (50-496)	*0.005*
U albumin (mg/l)	16.48 (1.44-434.24)	8.67 (2.14-234.65)	0.197

Values are expressed as mean value (or percent) and median value (5th-95th percentiles). SCr: serum creatinine; CCr: creatinine clearance; UCr: urea : creatinine ratio; UPCr: urine protein : creatinine ratio; UACr: urine albumin : creatinine ratio; Hgb: hemoglobin.

## Data Availability

The data used to support the findings of this study are included within the article.
